# Caries in children with and without orofacial clefting: A systematic review and meta‐analysis

**DOI:** 10.1111/odi.14183

**Published:** 2022-03-22

**Authors:** Rebecca E. Grewcock, Nicola P. T. Innes, Peter A. Mossey, Mark D. Robertson

**Affiliations:** ^1^ 3042 Dental Hospital and School University of Dundee Dundee UK; ^2^ 2112 School of Dentistry Cardiff University Cardiff UK

**Keywords:** children, cleft lip, cleft palate, dental caries, primary dentition, systematic review

## Abstract

This systematic review compared children's primary dentition caries experience for those with cleft lip and/or palate (CL/P) and without. Four databases were searched without date restriction for; cross‐sectional studies comparing caries experience for children with CL/P to those without. Screening, data extraction and risk assessment were carried out independently (in duplicate). Meta‐analyses used a random‐effects model. Twenty studies (21 reports) fitting the inclusion criteria comprised 4647 children in primary dentition from 12 countries. For dmft (*n* = 3016 children; 15 groups), CL/P mean = 3.2; standard deviation = 2.22 and no CL/P mean dmft = 2.5; sd 1.53. For dmfs (*n* = 1095 children; 6 groups), CL/P mean = 4; sd = 3.5 and no CL/P mean = 3; sd = 2.8. For % caries experience (*n* = 1094 children; 7 groups), CL/P mean = 65%; sd = 20.8 and no CL/P mean = 52%; sd = 28.1. Meta‐analysis showed higher caries experience in children with CL/P, standardised mean difference = 0.46; 95% CI = 0.15, 0.77. Studies' risk of bias was high (*n* = 7), medium (*n*−10) and low (*n* = 3). Children with CL/P had higher caries experience compared to those without CLP.

## 1 INTRODUCTION

Developmental anomalies of the mouth include clefting of the face and mouth. Orofacial clefts have been reported to have an incidence of 0.15% per live birth globally; however, it is understood that this figure varies across populations and may be associated with ethnic origin, genetic factors, environmental factors and gender (Mossey and Castillia, 2003; Mossey et al., 2009). These anomalies occur *in utero* because of incomplete fusion of the developing tissues and vary in extent (affecting combinations of the lip, alveolus and palate) and can be uni‐ or bi‐lateral. Cleft lip and/or palate (CL/P) can occur either in isolation, termed non‐syndromic CL/P, or in addition to other developmental syndromes (Shaw, 1993). As well as the aesthetic and functional problems they cause, there has been recent evidence that there are wider aspects of health and well‐being that affect those with CL/P (Ardouin et al., 2021). One of these is the experience of dental caries, which seems to be higher in those with CL/P than those without.

Early childhood caries carries with it a burden that can have significant and wide‐ranging consequences for the health and quality of life of children, including impaired cognitive development, poor school attendance and difficulty with schoolwork (Rebelo et al., 2019; Tsakos et al., 2013). A variety of possible reasons have been suggested. Not only are the reasons for a burden of additional dental caries experience for children with CL/P unclear, but the additional disease experience has not been quantified. Furthermore, it is understood that caries experience in the primary dentition leads to an undesirable dental disease trajectory throughout life (Hall‐Scullin et al., 2017), which may further compound oral health inequality in this group.

A systematic review and meta‐analysis on caries in CL/P patients was conducted by Antonarakis et al., 2013, nearly a decade ago (Antonarakis et al., 2013) and Worth et al. conducted a further systematic review and meta‐analysis on the same topic in 2017. Since 2017, there have been several primary research papers published on caries in CL/P warranting an update to the secondary literature (Chaudhari et al., 2021; Howe et al., 2017; Malay et al., 2021; Nagappan et al., 2019; Sunderji et al., 2017). Worth's review included 24 studies, two of which used national data for control groups that, while allowing for estimation of prevalence and direction of effect, may reduce confidence in the accuracy of the extent of any differences between groups. In addition, there has been some indication in the literature that the type and side of cleft might, respectively, influence the extent and location of caries.

Systematic review and meta‐analysis methodology is constantly improving and becoming more rigorous. Several more primary studies allowing for isolation of primary dentition caries experience have been carried out since the previous reviews' publication. In addition, no paper has looked at the care index (CI) and restorative index (RI) differences for children with and with no CL/P. This means that it is now timely to look at the question of quantification of dental caries disease burden and treatment carried out in the primary dentition of children with CL/P, compared with children with no CL/P using a systematic review of the scientific epidemiological and clinical literature, with meta‐analyses of the data where possible.

## 2 AIM AND OBJECTIVES

The aim was to investigate whether dental caries experience in the primary dentition of children with cleft lip and/or palate was different to that of children with no cleft lip and/or palate. The primary objective was to compare differences in caries rates in the primary dentition of children with cleft lip and/or palate (CL/P) to children without CL/P using dmft, or % caries experience, or other outcome measure, while the secondary objectives were to investigate whether there is an association between type of cleft and caries experience, and investigate the CI and RI between the CL/P and non‐CL/P groups.

## 3 METHODS

The protocol for this review was developed in accordance with Preferred Reporting Items for Systematic Review and Meta‐Analysis Protocols (PRISMA‐P) (Page et al., 2021). The review protocol was published in PROSPERO (https://www.crd.york.ac.uk/prospero/display_record.php?ID=CRD42021289287).

### 3.1 Searches

No date restrictions were imposed on database searches; however, papers published in languages other than English were excluded due to insufficient translation resources. The following databases were searched: PubMed (1946 to November 2021); Scopus (Elsevier interface, 1996 to November 2021); Web of Science (1900 to June 2017); and the Cochrane Library (CENTRAL, 1996 to 2021) to find relevant literature.

The following search strategy was designed and developed by the study authors with assistance from an Information Technologist at the University of Dundee. The search comprised Medical Subject headings (MeSH terms) and key text words appropriate to those children with cleft lip and/or palate (CL/P) and dental caries experience.

(Dental caries [MeSH] OR "dental caries" OR caries OR cavities OR "Tooth decay" OR "dental decay" OR plaque OR "oral health "oral hygiene" OR DMF OR DMFT) AND (Cleft Lip [MeSH] OR Cleft Palate [MeSH] OR "cleft lip" OR "cleft palate" OR "cleft lip and/or palate" OR "cleft lip and palate" OR CL/P OR "orofacial cleft" OR "oral cleft" OR cleft) AND (Child [MeSH] OR Child* OR preschool OR pediatric OR paediatric OR babies OR newborns OR infant*).

### 3.2 Inclusion criteria

#### 3.2.1 Participants/population

Children in the primary dentition with non‐syndromic CL/P and similar groups of children without CL/P.

#### 3.2.2 Study design

Observational and epidemiological primary research with data on caries experience collected through clinical examination were included. Studies that included permanent, primary and mixed dentitions were included; however, only primary dentition data were extracted. If it was not possible to extract data for the primary dentition in isolation, the study was excluded. Studies using national data as a comparator for the caries experience of children with CL/P were excluded.

### 3.3 Data management

#### 3.3.1 Study selection

**FIGURE 1:**
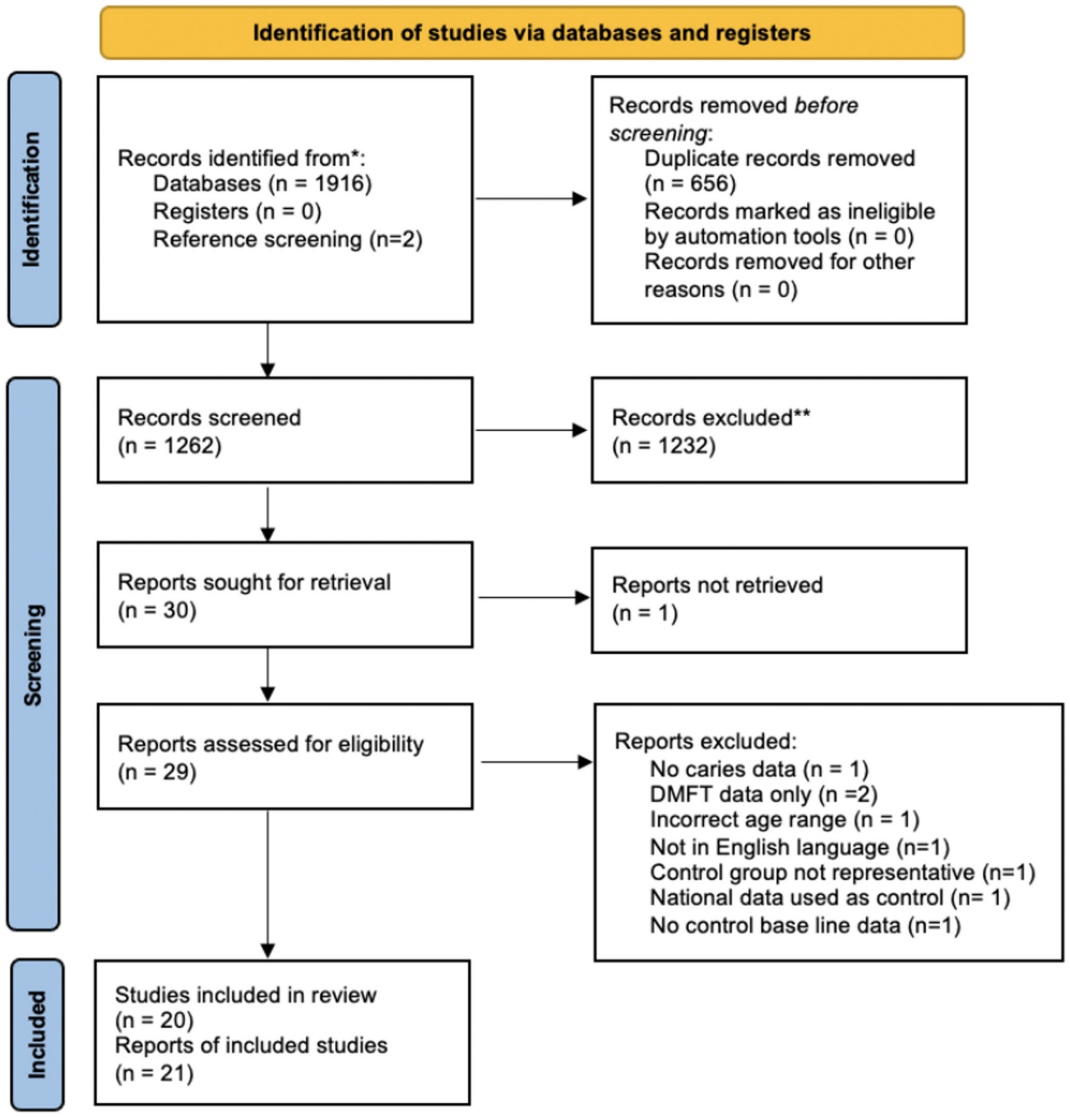
PRISMA flow diagram

Literature search results were entered into Microsoft Endnote software before completion of deduplication. Titles and abstracts were screened independently and in duplicate in Rayyan™ QCRI. Where there was disagreement on a study's eligibility for inclusion, consensus was achieved through discussion with a third reviewer. Following this, full texts were obtained and screened independently and in duplicate. Reviewers were not blinded to journal titles, study authors or institutions. The screening process was reported according to the Preferred Reported Items for Systematic Reviews and Meta‐Analysis (PRISMA) flow diagram (Figure 1) in addition to reasons for exclusion.

#### 3.3.2 Data extraction

Data extraction forms were developed and piloted by two authors. Piloting and subsequent discussion resulted in identification of required revisions and amendments, before extraction forms were re‐piloted. The forms following consensus and agreement between three authors (RG/MR/NI) were used to collect data independently and in duplicate by two investigators for data fields relating to the primary and secondary outcomes (RG/MR) and checked by a third (NI). A single investigator extracted data for the remaining fields relating to study characteristics, and a 10% randomly selected sample was extracted independently by one other investigator and cross‐checked to ensure there were no systematic data extraction errors. There were no systematic errors. The following data fields were extracted from included primary studies:

- Study characteristics
  - Author(s); Year of publication; Title; Aim(s) and objective(s); Study design: and Methodology
- Characteristics of data collection
  - Outcomes and outcome measures (caries experience tool used)
- Participant characteristics
  - Population description; Country; Age of participants; and Cleft Type
- Caries experience data
  - Quantitative caries experience data for the primary dentition (in the primary alone, mixed dentition alone where this was specified as relating to primary teeth and across the primary and mixed dentitions) for CL/P participants (and where available, for subgroups by cleft types) and no CL/P participants; Variance estimates (standard deviation); and Participants with no CL/P (control)
  - Data to allow calculation of the care‐ and restorative indices where possible.

#### 3.3.3 Data synthesis

Random‐effects meta‐analysis presented using standardised mean differences (SMD) was carried out for studies with adequate quantitative data with limited heterogeneity between collected caries experience data.

Mean dmft/deft/dmfs/dfs/ds caries experience for each study for children with CL/P and children with no CL/P was extracted or, where possible, calculated from the data presented. Studies that presented caries experience as a percentage of subjects within groups were excluded from meta‐analysis; instead, these data were extracted and described narratively and are presented in tables. Subgroup analyses were carried out for dentition types investigated (primary, primary and mixed together and mixed) where these data were available. Variance estimates were included in the meta‐analyses.

Standardised mean difference was used for caries experience across the groups which allowed the difference in size of caries experience between CL/P and no CL/P groups to be compared for different outcome measures (e.g. dmft and dmfs). Publication bias was evaluated using funnel plots. Asymmetric plots were considered to be an indication of publication bias.

#### 3.3.4 Risk of bias assessment

An adapted Newcastle‐Ottawa Scale (NOS) was used to assess and grade the quality of studies with a maximum score of ten points (five for ‘Selection’, two for ‘Comparability’ and three for ‘Outcome’) spread across seven domains (Appendix S1): The adapted NOS assessed the studies for sample representativeness of underlying populations; sample size; non‐respondents; comparability of outcome groups with controlled confounding factors; outcome assessment; and statistical testing. Scoring was conducted in duplicate, with a third reviewer to resolve any conflict. Studies were considered at low ROB when the overall scores are 7–8; medium ROB when scores are 4–6; and high ROB when they are 0–3 (Losilla et al., 2018).

## 4 RESULTS

### 4.1 Search results

Searching resulted in 1916 studies (1262 after deduplication). Following title and abstract screening, 30 full texts were checked. All potentially included studies, and all systematic reviews found during searching had references screened. Twenty studies met the inclusion criteria (Appendix S4).

### 4.2 Characteristics of studies

#### 4.2.1 Study designs

The studies were carried out between 1989 and 2021 and across 12 countries. Four studies were set in India (Chaudhari et al., 2021; Chopra et al., 2014; Malay et al., 2021; Nagappan et al., 2019), two in each of; Brazil (Tannure et al., 2012; Veiga et al., 2017), China (King et al., 2013; Zhu et al., 2010), Sweden (Sundell et al., 2016; Dahllof et al., 1989), UK (Ahluwalia et al., 2004; Lucas et al., 2000) and the United States (Howe et al., 2017; Sunderji et al., 2017), and there were single studies from Germany (Kirchberg et al., 2014), Greece (Parapanisiou et al., 2009), Ireland (Hewson et al., 2001), Netherlands (Bokhout et al., 1997), Jordan (Rawashdeh et al., 2011) and Thailand (Mutarai et al., 2008). Study design varied across the 20 studies with 15 using a case–control (Ahluwalia et al., 2004; Bokhout et al., 1997; Dahllof et al., 1989; Hewson et al., 2001; Howe et al., 2017; Lucas et al., 2000; Malay et al., 2021; Parapanisiou et al., 2009; Sundell et al., 2016; Sunderji et al., 2017; Tannure et al., 2012; Veiga et al., 2017; Zhu et al., 2010; King et al., 2013; Rawashdeh et al., 2011) and 5 using a matched comparative cross‐sectional design (Chaudhari et al., 2021; Chopra et al., 2014; Kirchberg et al., 2014; Mutarai et al., 2008; Nagappan et al., 2019).

### 4.3 Characteristics of participants

#### 4.3.1 Size of the participant groups

There were 4647 included in the studies, with data relating to the primary dentition. This comprised 2091 children in the CL/P group and 2,556 in the control, no CL/P group, with 3863 in the meta‐analyses (1655 CL/P; 2208 no CL/P). In the CL/P group, the number of participants ranged from 5 to 295 per study (mean = 103.95 sd = 89.80; median = 79) and for the no CL/P group participants per study ranged from 5 to 548 (mean = 123.65; sd = 135.31; median = 77.5).

#### 4.3.2 Participant ages

All studies reported on the caries status or primary teeth as this was one of the criteria for inclusion in the review.

Although only primary dentition data (or in some cases mixed dentition data) were collected, the participants were aged between 0 and 18 with a wide variety of age groups within this age bracket. There were nine studies that presented data from children within the age range from birth to 6 years and likely to have only primary teeth (Chopra et al., 2014; King et al., 2013; Kirchberg et al., 2014; Mutarai et al., 2008; Sunderji et al., 2017; Dahllof et al., 1989; Zhu et al., 2010; Sundell et al., 2016; Bokhout et al., 1996), six that presented data for children aged 5 years or over and likely to have caries data related to primary teeth as part of the mixed dentition phase of development (Ahluwalia et al., 2004; Chaudhari et al., 2021; King et al., 2013; Nagappan et al., 2019; Sundell et al., 2016; Zhu et al., 2010) and eight where dmft was presented although the age group crossed the primary and mixed dentitions (Hewson et al., 2001; Howe et al., 2017; Lucas et al., 2000; Malay et al., 2021; Parapanisiou et al., 2009; Sunderji et al., 2017; Tannure et al., 2012; Zhu et al., 2010) (Table 1). Three studies presented separate datasets for children in the primary dentition and mixed dentition stages (King et al., 2013; Sundell et al., 2016; Zhu et al., 2010).

**TABLE 1:** Dentition(s) studied, participant cohorts, and study ID

Primary dentition, mixed or both	Number of cohorts^a^	Study ID
Mixed	6	Ahluwalia et al., 2004; Chaudhari et al., 2021; King et al., 2013; Nagappan et al., 2019; Sundell et al., 2016; Zhu et al., 2010.
Primary	9	Chopra et al., 2014; King et al., 2013; Kirchberg et al., 2014; Mutarai et al., 2008; Sunderji et al., 2017; Dahllof et al., 1989; Zhu et al., 2010; Sundell et al., 2016; Bokhout et al., 1996.
Primary and mixed reported together	8	Hewson et al., 2001; Howe et al., 2017; Lucas et al., 2000; Malay et al., 2021; Parapanisiou et al., 2009; Sunderji et al., 2017; Tannure et al., 2012; Zhu et al., 2010.

^a^ Studies could be counted more than once.

#### 4.3.3 Caries assessment; examiners, recording indices and thresholds

There were eight different measures used for recording caries (Table 2) with some studies using more than one. The most commonly used outcome measures were dmft (*n* = 12) (Ahluwalia et al., 2004; Chopra et al., 2014; Hewson et al., 2001; King et al., 2013; Kirchberg et al., 2014; Lucas et al., 2000; Malay et al., 2021; Mutarai et al., 2008; Rawashdeh et al., 2011; Sunderji et al., 2017; Tannure et al., 2012; Zhu et al., 2010), caries presence (%) (*n* = 11) (Bokhout et al., 1996; Chaudhari et al., 2021; Chopra et al., 2014; Hewson et al., 2001; Howe et al., 2017; King et al., 2013; Mutarai et al., 2008; Parapanisiou et al., 2009; Sundell et al., 2016; Tannure et al., 2012; Zhu et al., 2010) and dmfs (*n* = 4) (Parapanisiou et al., 2009; Sundell et al., 2016; Veiga et al., 2017; Zhu et al., 2010). Fifteen studies used clinical examination alone (Bokhout et al., 1997; Chaudhari et al., 2021; Chopra et al., 2014; Hewson et al., 2001; Howe et al., 2017; King et al., 2013; Kirchberg et al., 2014; Lucas et al., 2000; Mutarai et al., 2008; Nagappan et al., 2019; Rawashdeh et al., 2011; Sundell et al., 2016; Tannure et al., 2012; Veiga et al., 2017; Zhu et al., 2010), three used both clinical examinations and radiographic assessment (Ahluwalia et al., 2004; Dahllof et al., 1989; Parapanisiou et al., 2009), and two used data from records (Malay et al., 2021; Sunderji et al., 2017).

**TABLE 2:** Caries outcome measure

Caries outcome measure	Number of studies	Study ID
dmft	12	Ahluwalia et al., 2004; Chopra et al., 2014; Hewson et al., 2001; King et al., 2013; Kirchberg et al., 2014; Lucas et al., 2000; Malay et al., 2021; Mutarai et al., 2008; Rawashdeh et al., 2011; Sunderji et al., 2017; Tannure et al., 2012; Zhu et al., 2010
Caries presence (%)	11	Bokhout et al., 1997; Chaudhari et al., 2021; Chopra et al.,^2014^; Hewson et al., 2001; Howe et al., 2017; King et al., 2013; Mutarai et al., 2008; Parapanisiou et al., 2009; Sundell et al., 2016; Tannure et al., 2012; Zhu et al., 2010
dmfs	4	Parapanisiou et al., 2009; Sundell et al., 2016; Veiga et al., 2017; Zhu et al., 2010
ICDAS	2	Chaudhari et al., 2021
deft	1	Nagappan et al., 2019
dfs	1	Dahllof et al., 1989
dft	1	Howe et al., 2017
ds	1	Dahllof et al., 1989

#### 4.3.4 Cleft type

Of the 20 studies (Table 3), five investigated caries experience by cleft type (Howe et al., 2017; Kirchberg et al., 2014; Mutarai et al., 2008; Sunderji et al., 2017; Zhu et al., 2010). Two studies detailed the caries experience for children with CL alone (Howe et al., 2017; Mutarai et al., 2008) and four for those with CP alone (Howe et al., 2017; Kirchberg et al., 2014; Mutarai et al., 2008; Zhu et al., 2010), while 17 studies gave no further information or breakdown by cleft type and one study stated that all participants had had UCL/P (Lucas et al., 2000). Two other studies included information on UCL/P and BCL/P (Mutarai et al., 2008; Sunderji et al., 2017). There was variation in the terms used to define cleft type.

**TABLE 3:** Cleft types included in primary studies

Study	CL	CP	CLP	UCLP	BCLP
Ahluwalia et al., 2004			✓		
Bokhout et al., 1996, 1997			✓		
Chaudhari et al., 2021			✓		
Chopra et al., 2014			✓		
Dahllof et al., 1989			✓		
Hewson et al., 2001			✓		
Howe et al., 2017	✓	✓	✓		
King et al., 2013			✓		
Kirchberg et al., 2014	✓	✓	✓		
Lucas et al., 2000				✓	
Malay et al., 2021			✓		
Mutarai et al., 2008	✓	✓		✓	✓
Nagappan et al., 2019			✓		
Parapanisiou et al., 2009			✓		
Rawashdeh et al., 2011			✓		
Sundell et al., 2016			✓		
Sunderji et al., 2017				✓	✓
Tannure et al., 2012			✓		
Veiga et al., 2017			✓		
Zhu et al., 2010	✓	✓			

#### 4.3.5 Non‐participation and representatives of the participant groups

##### Caries experience for children with CL/P compared to children with no CL/P

Table 4 shows summary data for each outcome measure with the underlying data from individual studies in Appendix S2. Caries experience scores were higher for all outcome measures apart from %dft.

**TABLE 4:** Data summary by outcome measure

	CL/P	No CL/P
dmft data (*n* = 15 groups of children from 13 studies)		
Total number of children (mean[sd]; median)	1318 (88 [64.5]; 71)	1698 (113 [130.3]; 69)
Mean dmft (sd)	3.2 (2.22)	2.5 (1.53)
dmfs data (*n* = 6 groups of children from 4 studies)		
Total number of children (mean[sd]; median)	480 (80 [32.1]; 79)	615 (103 [50.5]; 101)
Mean dmfs (sd)	4.1 (3.52)	3.2 (2.75)
% caries experience (*n* = 7 groups of children from 6 studies)		
Total number of children (mean[sd]; median)	567 (81 [42.1]; 76)	527 (75 [39.9]; 69)
Mean % caries experience	64.84	52.13
dft data (*n* = 1 group of children from 1 study)		
Number of children	76	75
Mean dft (sd)	0.59 (1.35)	0.11 (0.54)
dfs (*n* = 1 group of children from 1 study)		
Number of children	49	49
Mean dfs (sd)	7 (8.5)	3.9 (5.1)
%dft (*n* = 1 group of children from 1 study)		
Number of children	169	81
Mean dft	7.4	8.1

##### Meta‐analysis

Sixteen papers reported caries experience data (Bokhout et al., 1996/1997; Chopra et al., 2014; Dahllof et al., 1989; King et al., 2013; Kirchberg et al., 2014; Mutarai et al., 2008; Sundell et al., 2016; Sunderji et al., 2017; Tannure et al., 2012; Zhu et al., 2010; Hewson et al., 2001; Lucas et al., 2000; Rawashdeh et al., 2011; Veiga et al., 2017; Ahluwalia et al., 2004; Nagappan et al., 2019) where means and variance data were available. Of these, ten studies allowed isolation of the primary dentition data (Bokhout et al., 1996/1997; Chopra et al., 2014; Dahllof et al., 1989; King et al., 2013; Kirchberg et al., 2014; Mutarai et al., 2008; Sundell et al., 2016; Sunderji et al., 2017; Tannure et al., 2012; Zhu et al., 2010). Five studies provided data on the primary and mixed dentitions combined (Hewson et al., 2001; King et al., 2013; Lucas et al., 2000; Rawashdeh et al., 2011; Veiga et al., 2017), and four on the mixed dentition (Ahluwalia et al., 2004; Nagappan et al., 2019; Sundell et al., 2016; Zhu et al., 2010).

There were nine studies (Malay et al., 2021; Parapanisiou et al., 2009; Chopra et al., 2014; Mutarai et al., 2008; Zhu et al., 2010; Bokhout et al., 1996/1997; Chaudhari et al., 2021; King et al., 2013; Howe et al., 2017) with fourteen groups of children that could not be included in the meta‐analysis (MA) because there were no standard deviations included in the reports. Six reported %caries experience (Chopra et al., 2014; Mutarai et al., 2008; Zhu et al., 2010; Bokhout et al., 1997; Chaudhari et al., 2021; King et al., 2013), Howe et al., 2017 used a % dft, Malay et al., 2021 and Parapanisiou et al., 2009 used dmft and dmfs data, respectively, with no variance data. Although these data were not included in the meta‐analysis, they were included in summary data presented in Table 4.

**FIGURE 2:**
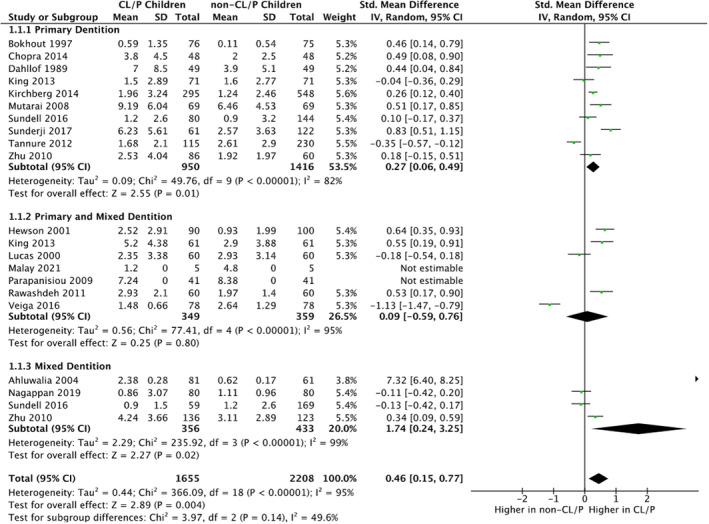
Forrest plot as part of random‐effects meta‐analysis portraying caries experience of CL/P children compared to non‐CL/P children in: 1.1.1 the primary dentition; 1.1.2 the primary and mixed dentition; and 1.1.3 the mixed dentition presented as standardised mean differences for dmft/DMFT and 95% confidence intervals (CI). Each subgroup has a black diamond to illustrate the point estimate and CI of effect, with the final diamond illustrating the overall difference in caries experience between the CL/P and non‐CL/P children

A random‐effects MA was run due to high study heterogeneity (*I* ^2^ = 82% primary dentition, 95% primary and mixed dentitions combined, and 99% mixed dentition). Meta‐analysis found statistically significant difference in the caries experience of CL/P and non‐CL/P in; the primary dentition (SMD = 0.27; 95% CI = 0.06, 0.49); and the mixed dentition (SMD = 1.74 95% CI = 0.24, 3.25). Meta‐analysis found no evidence of a difference in the combined primary and mixed dentitions (SMD = 0.09 95% CI = −0.59, 0.76). The overall effect across all dentitions combined demonstrated a statistically significant difference in caries experience between CL/P and non‐CL/P with CL/P children experiencing a higher caries burden (SMD = 0.46 95% CI = 0.15–0.77), *p* = 0.004 (Figure 2).

##### Assessment of publication bias

**FIGURE 3:**
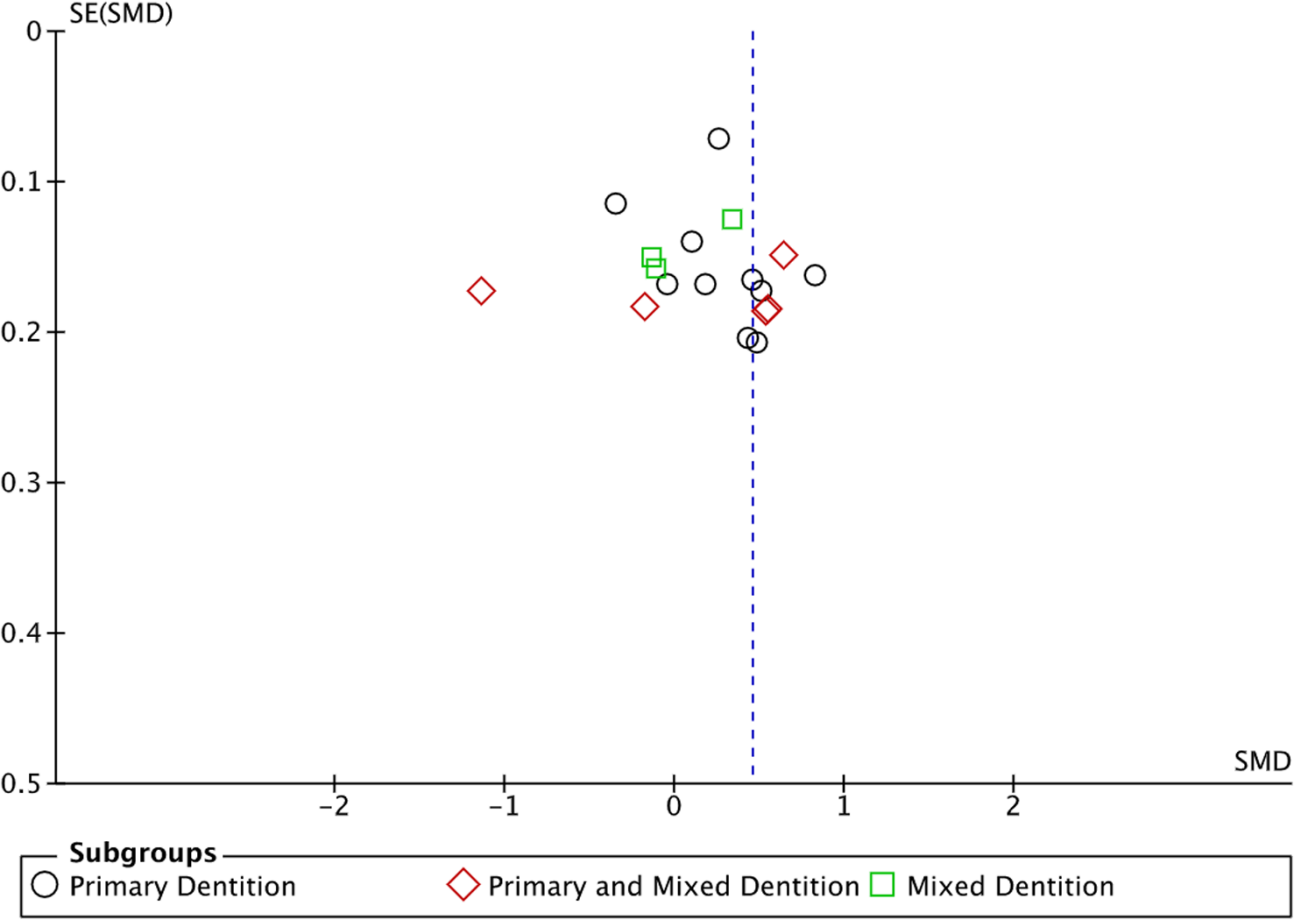
Funnel plot as part of random‐effects meta‐analysis to investigate publication bias across primary studies

Publication bias was assessed as part of meta‐analysis using a funnel plot (Figure 3) and Egger's regression intercept test. Asymmetry with respect to the *x*‐axis' overall effect indicates a possible presence of publication bias across primary studies.

##### Risk of bias assessment (ROB)

The quality and risk of bias of included studies were assessed using an adapted Newcastle‐Ottawa Scale (NOS) (Appendix S3), with a numerical score was awarded to each study. Risk of bias scoring ranged from two to seven out of a possible eight across three domains, with a mean score of 4.45 indicating a generally medium risk of bias across the studies. The mean score of each domain was calculated: 1.09 out of a possible 3 for *Selection*; 1.30 out of a possible 2 for *Comparability*; and 1.50 out of a possible 3 for outcome. Of the 20 studies in this systematic review, a summary across the studies is shown below:

- High ROB (scoring 0–3 out of a possible 8, across three domains); *n* = 7 (Chaudhari, 2021; Chopra, 2014; Lucas, 2000; Malay, 2021; Nagappan, 2019; Parapanisiou et al., 2009; Veiga et al., 2017).
- Medium ROB (scoring 4–6 out of a possible 8, across three domains); *n* = 10 (Ahluwalia et al., 2004; Bokhout et al., 1997; Dahllof et al., 1989; Hewson et al., 2001; King et al., 2013; Mutarai et al., 2008; Rawashdeh et al., 2011; Sunderji et al., 2017; Tannure et al., 2012; Zhu et al., 2010).
- Low ROB (scoring 7–8 out of a possible 8, across three domains); *n* = 3 (Howe, 2017; Kirchberg, 2014; Sundell et al., 2016).

##### Care index (CI) and restorative index (RI)

Five studies provided some component data of caries measurement indices (Dahllof et al., 1989; Howe, 2017; King et al., 2013; Nagappan, 2019, Veiga, 2016). This was only available for the primary dentition. Three studies reported decayed, missing and filled data across groups allowing for calculation of the CI (f/d+m+f) and RI (f/d+f) (King et al., 2013; Nagappan, 2019; Veiga, 2016), while two studies reported decayed and filled data allowing for calculation of the RI only (Dahllof et al., 1989; Howe, 2017) (Table 5).

**TABLE 5:** Care and restorative indices (CI and RI) for CL/P and non‐CL/P children

Study	CL/P CI	No CL/P CI	CL/P RI	No CL/P RI
Dahllof et al., 1989	–	–	0.31	0.62
Howe 2017	–	–	0.31	0.04
King et al., 2013 (2–4 years)	0.40	0.06	0.40	0.06
King et al., 2013 (5–7 years)	0.65	0.18	0.69	0.19
Nagappan 2019	0.03	0.33	0.03	0.35
Veiga 2016	0.30	0.42	0.36	0.43

Of the five studies for which the RI could be calculated, three of them (Dahllof et al., 1989; Nagappan, 2019; Veiga, 2016) showed a lower RI for children with CL/P compared to children with no CL/P demonstrating a lower proportion of restoratively managed carious teeth. Two out of three studies for which the CI could be calculated (Nagappan, 2019; Veiga, 2016) showed a higher CI for non‐CL/P children demonstrating a higher proportion of extraction‐based treatment in CL/P children compared to non‐CL/P children.

## 5 DISCUSSION

This systematic review and meta‐analysis found that children with a cleft lip and/or palate have an increased caries experience compared to their non‐cleft counterparts in the primary dentition (SMD = 0.46; 95% CI = 0.15, 0.77). Twelve studies included in this review were published in the last decade (Chaudhari et al., 2021; Chopra et al., 2014; Howe et al., 2017; Kirchberg et al., 2014; Malay et al., 2021; Nagappan et al., 2019; Sundell et al., 2016; Sunderji et al., 2017; Tannure et al., 2012; Veiga et al., 2017; King et al., 2013; Rawashdeh et al., 2011), five (Chaudhari et al., 2021; Malay et al., 2021; Nagappan et al., 2019; Howe et al., 2017; Sunderji et al., 2017) of which were published following the last systematic review (Worth et al., 2017), providing rationale for an updated study.

The standardised mean difference (SMD) was used in the meta‐analysis as this allows different scales used to measure caries (dmft, dmfs, etc.) to be compared directly. SMD uses a ratio between the outcomes to allow the populations (children with CL/P and those without) to be compared. While SMDs are not intuitive to interpret and are limited in their ability to tell us the actual value of the difference between two populations, they allow us to measure the strength of evidence against the null hypothesis of no difference between them. The linear scale forest plot shows the sample estimates for each trial, the subgroup analyses and overall analyses (together with their 95% confidence intervals) symmetrically around the line of no difference. We only extracted data that specified the primary dentition so dmfs/dmft/% caries experience in the primary detention (or the corresponding age group). We subgrouped the unique primary tooth caries experience data, by the child's age, into the 10 studies relating only to the primary dentition which showed a difference between the two populations and higher caries incidence in the CL/P group (SMD = 0.27; 95% CI = 0.06, 0.49), the four covering both the primary and mixed dentition which showed no evidence of a difference (0.09; −0.59, 0.76) and the five with data on primary tooth caries experience in the mixed dentition which again showed a difference with caries experience being higher again the CL/P group (1.74; 0.24, 3.25).

There have been several previous systematic reviews which have also shown a positive relationship between increased caries rates and children with orofacial clefts.

The most recent review was by Worth et al, 2017 where 17 studies with primary teeth were included. However, we excluded five of the studies they included because they were considered to be at high risk of overlapping data with another included study (Hazza'a et al., 2011), compared with national data rather than a directly comparable group (Britton and Welbury, 2010) were not in English language (Hochstein and Hochstein, 1970; Bethmann et al., 1967; Bethmann et al., 1968) or included children likely to be in the permanent dentition (Pisek et al., 2014).

Only five studies allowed the CI and/or the RI for children with CL/P and those with no CL/P to be calculated. Although there was limited data, and this was disproportionately available for the few studies that found children with CL/P to have less caries, it was still apparent that children with no CL/P were more likely to receive interventive management of dental caries. In addition, when that intervention was delivered, for children with CL/P, they were more likely to receive extraction‐based treatment compared to children with no CL/P who were more likely to have restorative‐based treatment. This finding is similar to a systematic review of children with and without learning disabilities (Robertson et al., 2019). However, the majority of studies in this review did not provide component data of caries measurement indices; therefore, caution should be exercised in the interpretation of these data as it is not representative of all included studies.

There are a number of putative factors that might contribute to the increased caries risk. Across global epidemiological data sets, children with CL/P are generally born into more socioeconomically deprived areas than those children without CL/P (Chung et al., 2019; Swanson et al., 2017). Many parents/caregivers of children with CL/P have a reduced oral health literacy and poor engagement with healthcare professionals, inadequate home‐delivered oral hygiene practices and increased consumption of dietary fermentable carbohydrates (Baskaradoss, 2018). Furthermore, certain risk factors may contribute to the correlation between CL/P and increased caries experience including feeding practice differing between children with CL/P and those without, with CL/P often having extended periods of bottle‐feeding; with a greater sugar content compared to breast‐feeding (Lin and Tsai, 1999). Individuals with CL/P have been found to have a reduced salivary flow with normal salivary flow rates in 55% children with CL/P (0.7 ml/min) compared to 66% of non‐cleft children (Parapanisiou et al., 2009). Mouth breathing is common in those with CL/P (Halitchi et al., 2017; Hazza'a et al., 2011; Tuaño‐Cabrera et al., 2017) and considered a risk modifier to dental caries due to the drying effect it has on the oral cavity and reducing salivary flow. Finally, there may be an association between the anatomical features of the repaired cleft where scar tissue and dental irregularity can affect access to parts of the oral cavity and result in prolonged retention of residual food.

The possibility that cleft areas are associated with areas of stagnation, and the promotion of dysbiotic plaque biofilms, is supported by several observations: the presence of a fistula (Richards et al., 2015); the severity of the cleft (Lehtonen et al., 2015; Mian et al., 2005); and untreated clefts (Kamble et al., 2017) have been positively associated with increased caries. As such, oral health promotion measures in these patients must be prioritised by clinical teams to mitigate against the increased caries risk, and caries incidence, experienced by those patients with CL/P; early access to dental care may help to preclude dental caries, and associated treatments, in childhood and into adulthood.

Twenty studies were included in this systematic review and meta‐analysis. There was significant inter‐study heterogeneity of data across domains, especially with respect to outcome reporting and outcome measures. Variation existed among study methodologies and measurements criteria used to assess caries experience. Three studies used radiographs in addition to clinical examination to detect caries, improving the accuracy of diagnosis (Parapanisiou et al., 2009; Dahllof et al., 1989; Ahluwalia et al., 2004).

A limitation of this review is that only studies published in the English language were included, due to a lack of resources available to bring about high‐quality translation. However, there was no restriction on country of origin.

Assessing studies' risk of bias, using a modified Newcastle‐Ottawa Scale (NOS), it was found that the majority of studies were of medium risk of bias, followed by high risk of bias, with only 3 studies having a low risk of bias (Howe, 2017; Kirchberg et al., 2014; Sundell et al., 2016). The quality of the studies included should be considered and the influence this has on the concluding outcomes of this systematic review.

The methodology used in this systematic review and meta‐analysis is a widely accepted, and standard practice. However, it is important to appraise the methodology used in this systematic review for its overarching strengths and limitations.

Ten of 20 included studies were from developing countries (Tannure et al., 2012; King et al., 2013; Chaudhari et al., 2021; Chopra et al., 2014; Malay et al., 2021; Nagappan et al., 2019; Veiga et al., 2017; Mutarai et al., 2008; Rawashdeh et al., 2011; Zhu et al., 2010), and 10 from developed countries (Dahllof et al., 1989; Lucas et al., 2000; Ahluwalia et al., 2004; Bokhout et al., 1997; Parapanisiou et al., 2009; Sundell et al., 2016; Kirchberg et al., 2014; Hewson et al., 2001; Howe et al., 2017; Sunderji et al., 2017): in accordance with the United Nations (2020) ‘World Economic Situation & Prospects’ (York UNN, 2020)—a fair distribution of studies from both the developed and developing world. Given the known influence of socioeconomic status on the access to oral and dental care, the inclusion of studies from developing countries is essential in order to provide a representative global depiction and is therefore a strength of this review.

Sample sizes between CL/P and control groups were equally matched across eleven studies (Chopra et al., 2014; King et al., 2013; Mutarai et al., 2008; Lucas et al., 2000; Malay et al., 2021; Rawashdeh et al., 2011; Nagappan et al., 2019; Parapanisiou et al., 2009; Vegia et al., 2016; Chaudhari et al., 2021; Dahllof et al., 1989), which is a strength in the representativeness of both studied participant groups.

## 6 CONCLUSIONS

The overall findings of the meta‐analyses align with previous global literature, showing higher caries experience in children with CL/P compared to those without, for children's primary and mixed dentition caries experience. This translates into additional burden of morbidity and need for care, over and above the care needs as a result of the orofacial clefting. Additionally, the data indicate that children with CL/P may receive less restorative treatment for dental caries than non‐CL/P children, and be provided with more extraction‐based treatment.

## 7 IMPLICATIONS FOR FUTURE RESEARCH

Following this thorough systematic review of caries in CLP which included five studies in the last 3 years, some notable gaps in the literature were detected, and this will help inform future work in this field. A subgroup analysis comparing CP and CL/P as different entities was not possible due to the dearth of data on isolated CP. Likewise additional evidence for the extent to which caries experience in the permanent dentition in those with CL/P would provide a more complete picture of residual morbidity and oral health‐related quality of life due to dental caries.

Another area for future research attention is further enquiry into the aetiology of caries in CL/P with an explanation of why there is a consistently greater caries experience among this marginalised group, and this would inform the preventative strategies to combat the problem. From current evidence, it is likely that the causes are a combination of biological and environmental factors, and there are newly emerging methods for behaviour change that could be employed to address the problem.

Future clinical trials in this field would benefit from the provision of a core set of outcomes informing risk factors for the initiation and progression of dental caries in CL/P children, while also defining these outcomes. There are now three systematic reviews in this field, and defined outcome sets would assist trialists and systematic reviewers in pooling outcomes with respect to methodological design and homogeneity of data. Lastly, the literature would benefit from future work in which the contributing components of dmft/DMFT are presented, facilitating derivation of more comprehensive and representative CI and RI for children with CL/P and control groups. This may help in future training of dental professionals in terms of their clinical management of children with CL/P and with resource allocation.

## CONFLICT OF INTEREST

No conflicts to declare.

## AUTHOR CONTRIBUTIONS

**Rebecca E Grewcock:** Conceptualization; Data curation; Formal analysis; Investigation; Methodology; Writing – original draft; Writing – review & editing. **Nicola PT Innes:** Conceptualization; Data curation; Formal analysis; Investigation; Methodology; Project administration; Supervision; Validation; Visualization; Writing – original draft; Writing – review & editing. **PA Mossey:** Conceptualization; Data curation; Formal analysis; Investigation; Methodology; Project administration; Supervision; Validation; Visualization; Writing – original draft; Writing – review & editing. **Mark D Robertson:** Conceptualization; Data curation; Formal analysis; Investigation; Methodology; Project administration; Software; Supervision; Validation; Writing – original draft; Writing – review & editing.

### PEER REVIEW

The peer review history for this article is available at https://publons.com/publon/10.1111/odi.14183.

## Supporting information

App S1

App S2

App S3

App S4
