# Supplementary figures and images for: Caries in children with and without orofacial clefting: A systematic review and meta‐analysis
Source: Oral Dis. 2022 Mar 22;28(5):1400–11. doi: 10.1111/odi.14183 (PMC9314085; doi:10.1111/odi.14183)

Appendix 1: Adapted Newcastle-Ottawa Scale (NOS)


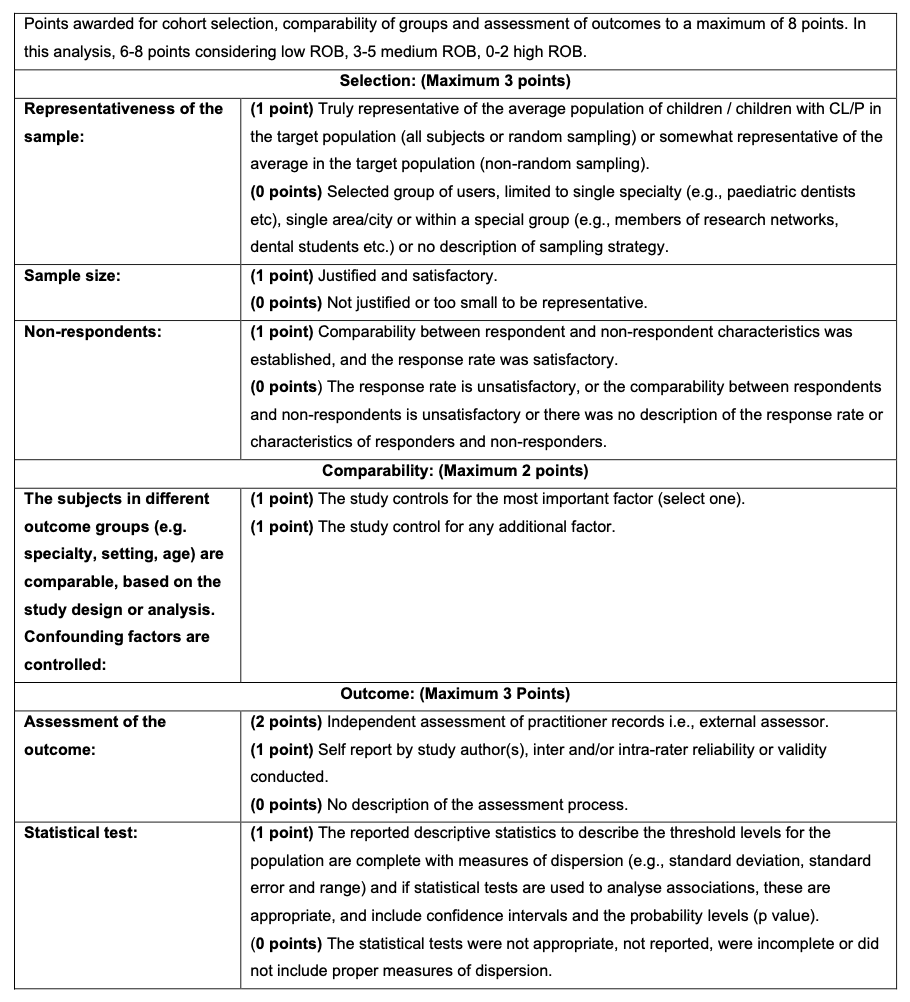

Supplement: Supplementary file 1 — App S1 [file ODI-28-1400-s004.docx]
