# Supplementary material for: Caries in children with and without orofacial clefting: A systematic review and meta‐analysis
Source: Oral Dis. 2022 Mar 22;28(5):1400–11. doi: 10.1111/odi.14183 (PMC9314085; doi:10.1111/odi.14183)
Supplement: Supplementary file 2 — App S2 [file ODI-28-1400-s001.docx]

Appendix 2. Data table for individual studies and groups by outcome measure and dentition.

|  | | no of participants | |  | **dmft** | | | | dentition by age group |
| --- | --- | --- | --- | --- | --- | --- | --- | --- | --- |
| Study no | **Study name** | **CLP** | **No CL/P** | **Ages (years)** | **CLP mean dmft** | **CLP sd** | **No CL/P mean dmft** | **No CLP sd** |  |
| 5 | Chopra | 48 | 48 | 4-6 | 3.8 | 4.5 | 2 | 2.5 | primary |
| 22 | King (2-4) | 71 | 71 | 2-4 | 1.5 | 2.89 | 1.6 | 2.77 | primary |
| 9 | Kirchberg | 295 | 548 | 1-6 | 1.96 | 3.24 | 1.24 | 2.46 | primary |
| 12 | Mutarai | 69 | 69 | 1.5-3 | 9.19 | 6.04 | 6.46 | 4.53 | primary |
| 17 | Sunderji | 61 | 122 | 2-6 | 6.23 | 5.61 | 2.57 | 3.63 | primary |
| 18 | Tannure | 115 | 230 | primary (4+) | 1.68 | 2.1 | 2.61 | 2.9 | primary |
| 21 | Zhu (3-5) | 86 | 60 | 3-5 | 2.53 | 4.04 | 1.92 | 1.97 | primary |
| 7 | Hewson | 90 | 100 | 1.5- 17 | 2.52 | 2.91 | 0.93 | 1.99 | primary/mixed |
| 22 | King (5-7) | 61 | 61 | 5-7 | 5.2 | 4.38 | 2.9 | 3.88 | primary/mixed |
| 10 | Lucas | 60 | 60 | 3-15 | 2.35 | 3.38 | 2.93 | 3.14 | primary/mixed |
| 11 | Malay | 5 | 5 | 3-17 | 1.2 | No data | 4.8 | No data | primary/mixed |
| 23 | Rawashdeh | 60 | 60 | 0-17 | 2.93 | 2.1 | 1.97 | 1.4 | primary/mixed |
| 1 | Ahluwalia | 81 | 61 | 6-16 | 2.38 | 0.28 | 0.62 | 0.17 | mixed |
| 13 | Nagappan | 80 | 80 | 8-16 | 0.86 | 3.07 | 1.11 | 0.96 | mixed |
| 21 | Zhu (6-12) | 136 | 123 | 6-12 | 4.24 | 3.66 | 3.11 | 2.89 | mixed |
|  | Total | 1318 | 1698 |  |  |  |  |  |  |
|  | mean | 88 | 113 |  | 3.2 |  | 2.5 |  |  |
|  | sd | 64.5 | 130.3 |  | 2.22 |  | 1.53 |  |  |
|  | median | 71.0 | 69.0 |  |  |  |  |  |  |
|  | | no of participants | |  | **dmfs** | | | |  |
| Study no | **Study name** | **CLP** | **No CL/P** | **Ages (years)** | **CLP mean dmfs** | **CLP sd** | **no CL/P mean dmfs** | **no CLP sd** |  |
| 16 | Sundell (5yrs) | 80 | 144 | 5 | 1.2 | 2.6 | 0.9 | 3.2 | primary |
| 21 | Zhu (3-5) | 86 | 60 | 3-5 | 4.38 | 9.25 | 2.28 | 2.3 | primary |
| 14 | Parapanisiou | 41 | 41 | 4-18 | 7.24 | No data | 8.38 | No data | primary/mixed |
| 19 | Veiga | 78 | 78 | 5-18 | 1.48 | 0.66 | 2.64 | 1.29 | primary/mixed |
| 16 | Sundell (10yrs) | 59 | 169 | 10 | 0.9 | 1.5 | 1.2 | 2.6 | mixed |
| 21 | Zhu (6-12) | 136 | 123 | 6-12 | 9.26 | 10.06 | 3.98 | 3.83 | mixed |
|  | Total | 480 | 615 |  |  |  |  |  |  |
|  | mean | 80 | 103 |  | 4 |  | 3 |  |  |
|  | sd | 32.1 | 50.5 |  | 3.5 |  | 2.8 |  |  |
|  | median | 79 | 100.5 |  |  |  |  |  |  |
|  | | no of participants | |  | **caries experience (%)** | | | |  |
| Study no | **Study name** | **CLP** | **No CL/P** | **Ages (years)** | **CLP %** | **CLP sd** | **no CL/P %** | **no CLP sd** |  |
| 5 | Chopra | 48 | 48 | 4-6 | 71.9 | no data | 60.9 | no data | primary |
| 12 | Mutarai | 69 | 69 | 1.5-3 | 91.3 | no data | 89.9 | no data | primary |
| 21 | Zhu (3-5) | 86 | 60 | 3-5 | 70.9 | no data | 66.9 | no data | primary |
| 3 | Bokhout | 76 | 75 | 2-4 | 30.9 | no data | 6.5 | no data | primary |
| 4 | Chaudhary | 20 | 20 | 5-12 | 60 | no data | 30 | no data | primary/mixed |
| 22 | King | 132 | 132 | 2-7 | 46.5 | no data | 40.8 | no data | primary/mixed |
| 21 | Zhu (6-12) | 136 | 123 | 6-12 | 82.4 | no data | 69.9 | no data | mixed |
|  | Total | 567 | 527 |  |  |  |  |  |  |
|  | mean | 81 | 75 |  | 64.84 |  | 52.13 |  |  |
|  | sd | 42.1 | 39.9 |  |  |  |  |  |  |
|  | median | 76 | 69 |  |  |  |  |  |  |
|  |  |  |  |  |  |  |  |  |  |
|  |  | no of participants | |  | **dft** | | | |  |
| Study no | **Study name** | **CLP** | **No CL/P** | **Ages (years)** | **CLP mean dft** | **CLP sd** | **no CL/P mean dft** | **no CLP sd** |  |
| 3 | Bokhout | 76 | 75 | 2-4 | 0.59 | 1.35 | 0.11 | 0.54 | primary |
|  |  |  |  |  |  |  |  |  |  |
|  |  | no of participants | |  | **dfs** | | | |  |
| Study no | **Study name** | **CLP** | **No CL/P** | **Ages (years)** | **CLP mean dfs** | **CLP sd** | **no CL/P mean dfs** | **no CLP sd** |  |
| 6 | Dahllof | 49 | 49 | 5 | 7 | 8.5 | 3.9 | 5.1 | primary |
|  |  |  |  |  |  |  |  |  |  |
|  |  | no of participants | |  | **% dft** | | | |  |
| Study no | **Study name** | **CLP** | **No CL/P** | **Ages (years)** | **CLP mean dft** | **CLP sd** | **no CL/P mean dft** | **no CLP sd** |  |
| 8 | Howe | 169 | 81 | 3 | 7.4 | No data | 7.1 | No data | primary |
