# Supplementary material for: Caries in children with and without orofacial clefting: A systematic review and meta‐analysis
Source: Oral Dis. 2022 Mar 22;28(5):1400–11. doi: 10.1111/odi.14183 (PMC9314085; doi:10.1111/odi.14183)
Supplement: Supplementary file 3 — App S3 [file ODI-28-1400-s002.docx]

Appendix 3 Newcastle Ottawa Scale results

| Study name | **Newcastle Ottawa Scale Assessment** | | | | | | Total (out of 8) |
| --- | --- | --- | --- | --- | --- | --- | --- |
|  | Selection | | | Comparability | Outcome | |  |
|  | Representativeness of sample | Sample Size | Non respondent | Subjects in different outcomes | Assessment of outcome | Stat test |  |
| Ahluwalia | 1 | 1 | 0 | 1 | 1 | 1 | **5** |
| Tannure | 1 | 1 | 0 | 1 | 1 | 1 | **5** |
| Veiga | 0 | 0 | 0 | 2 | 0 | 1 | **3** |
| Zhu | 1 | 1 | 0 | 2 | 1 | 1 | **6** |
| King | 1 | 1 | 0 | 1 | 1 | 1 | **5** |
| Kirchberg | 1 | 1 | 1 | 2 | 1 | 1 | **7** |
| Parapanisiou | 0 | 0 | 0 | 1 | 1 | 1 | **3** |
| Chaudhari | 0 | 0 | 0 | 1 | 1 | 1 | **3** |
| Chopra | 0 | 0 | 0 | 1 | 1 | 1 | **3** |
| Malay | 0 | 0 | 0 | 1 | 1 | 1 | **3** |
| Nagappan | 0 | 0 | 0 | 1 | 1 | 1 | **3** |
| Hewson | 1 | 1 | 0 | 1 | 1 | 1 | **5** |
| Rawashdeh | 0 | 0 | 0 | 1 | 2 | 1 | **4** |
| Mutarai | 0 | 0 | 0 | 2 | 1 | 1 | **4** |
| Dahllof | 0 | 0 | 1 | 1 | 1 | 1 | **4** |
| Sundell | 1 | 1 | 1 | 2 | 1 | 1 | **7** |
| Bokhout | 1 | 1 | 0 | 1 | 1 | 1 | **5** |
| Lucas | 0 | 0 | 0 | 1 | 1 | 1 | **3** |
| Howe | 1 | 1 | 1 | 2 | 1 | 1 | **7** |
| Sunderji | 0 | 0 | 0 | 2 | 1 | 1 | **4** |
