# Supplementary material for: Caries in children with and without orofacial clefting: A systematic review and meta‐analysis
Source: Oral Dis. 2022 Mar 22;28(5):1400–11. doi: 10.1111/odi.14183 (PMC9314085; doi:10.1111/odi.14183)
Supplement: Supplementary file 4 — App S4 [file ODI-28-1400-s003.docx]

Appendix 4: summary of included primary studies

|  | | | | **Participant numbers** | |  |  |  |
| --- | --- | --- | --- | --- | --- | --- | --- | --- |
| **Author** | **Year** | **Country** | **Study Design** | **Cleft** | **Non-cleft** | **Age (years)** | **Main Findings** | **Total ROB (out of 8)** |
| Ahluwalia | 2004 | UK | Case Control | 81 | 61 | 6-16 | The longer clearance times of foods and consequent generation of fermentable sugars from starches may contribute to higher caries prevalence observed in children with cleft palates. | 5 |
| Bokhout | 1997/96 | The Netherlands | Case Control | 76 | 75 | 4 | Children with an oral cleft are at higher risk of dental caries. Children with an oral cleft and their parents should be subjected as early as possible to a preventive programme that aims at preserving a healthy primary dentition. Special attention should be paid to oral hygiene in the cleft area. | 5 |
| Chaudhari | 2021 | India | Cross-sectional | 20 | 20 | 5-12 | Children with CL/P showed limited access to caries protective measures and low buffering capacity in resting saliva, along with elevated levels of salivary Streptococci and Lactobacilli in stimulated saliva. | 3 |
| Chopra | 2014 | India | Cross-sectional | 74 | 8 | 4-6 | Differences of oral health status exist among 4-6 year old children with and without clefts. Children with clefts fare worse in terms of dental caries, gingival health, oral mucosal health and malocclusion. | 3 |
| Dahllof | 1989 | Sweden | Case Control | 49 | 49 | 5 | Children with clefts can be considered as a group having an increased risk of caries. Several factors, such as early establishment of proximal contacts and increased incidence of enamel hypomineralisation, may contribute to increased risk of caries. The dentist is responsible for initiating the preventative program and should be included in the cleft team. | 4 |
| Hewson | 2001 | Ireland | Case Control | 90 | 100 | 1.5-18 | Significantly greater risk of dental disease in clefting, particularly in primary dentition. | 5 |
| Howe | 2017 | USA | Case Control | 1138 | 2687 | 3-18 | The seemingly higher risk for dental decay associated with increased dental anomalies may be superseded by possible greater access to dental care. | 7 |
| King | 2013 | China | Case Control | 132 | 132 | 2-4 & 5-7 | The 5- to 7-year-old children with CLP had a higher caries experience in the primary dentition than the children without CLP | 5 |
| Kirchberg | 2014 | Germany | Cross-sectional | 295 | 548 | 1-6 | Greater caries prevalence in CLP children than controls. Children with less severe clefts had a level of caries that is comparable to children from the general population. | 7 |
| Lucas | 2000 | UK | Case Control | 60 | 60 | 3-15 | Dental health of children with UCLP was found to be similar to a group matched control. These children are cared for in a multidisciplinary centre and received preventive dental care from a very early age. Although there was no significant difference in dmfs, the children with UCLP had a greater number of unrestored cavities, compared with the control children, demonstrating a need for more effective coordinated restorative dental services for these children. | 3 |
| Malay | 2021 | India | Case Control | 5 | 5 | 3-17 | Children with cleft lip and palate showed lesser incidences of dental caries when compared to children without cleft lip and palate. | 3 |
| Mutarai | 2008 | Thailand | Cross-sectional | 69 | 69 | 1.5-3 | The prevalence of early childhood caries was considerably high in southern Thai cleft children aged 18-36 months and confirmed previous reports of a significantly greater caries experience than in non cleft children. | 4 |
| Nagappan | 2019 | India | Cross-sectional | 80 | 80 | 8-16 | Cleft children have a reduced oral health-related quality of life compared to non-cleft children | 3 |
| Parapanisiou | 2009 | Greece | Case Control | 41 | 41 | 4-18 | Increased numbers of initial/ white spot lesions combined with poor OH found in a Greek group of children with CLP predisposes to an increased risk for further development of carious cavitated lesions. The application of an intensive individualised OH preventive program focused on improvement of OH and remineralisation of whit spot lesions is imperative. | 3 |
| Rawashdeh | 2011 | Jordan | Case Control | 60 | 60 | 0-17 | Patients with clefts had a significantly higher rate of oral candidal colonisation compared with control subjects, which varied with age, type of cleft, and the number of surgical interventions. Oral health status was significantly poorer in patients with clefts. | 4 |
| Sundell | 2016 | Sweden | Case Control | 139 | 313 | 5 & 10 | Preschool children with cleft lip and/or palate seem to have more caries in the primary dentition than age-matched non-cleft controls. Enamel defects were more common in CL(P) children in both age groups. | 7 |
| Sunderji | 2017 | USA | Case Control | 61 | 122 | 2-6 | Children with complete CLP are at greater risk of anterior and posterior caries. No significant difference between UCLP and BCLP | 4 |
| Tannure | 2012 | Brazil | Case Control | 115 | 230 | 4-21 | Caries experience in children born with clefts is not higher in comparison to control children. | 5 |
| Veiga | 2016 | Brazil | Case Control | 78 | 78 | 5-18 | Caries experience in both primary and permanent dentitions was higher for individuals without CL/P. Poor OH and presence of gingival inflammation were more evident in subjects with CLP. | 3 |
| Zhu | 2010 | China | Case Control | 380 | 339 | 3-15 & 6-12 | Subjects with CL/P are at a significant risk of caries, especially in maxillary anterior teeth. Patients and their families are preoccupied with other aspect of their health e.g., surgery, nutrition, mental health, and speech development. | 6 |
